# Mechanisms of Non-alcoholic Fatty Liver Disease and Beneficial Effects of Semaglutide: A Review

**DOI:** 10.7759/cureus.67080

**Published:** 2024-08-17

**Authors:** Sultan Alfawaz, Abdulhadi Burzangi, Ahmed Esmat

**Affiliations:** 1 Department of Clinical Pharmacology, King Abdulaziz University, Faculty of Medicine, Jeddah, SAU

**Keywords:** insulin resistance, glp-1 receptor agonist, type 2 diabetes mellitus, nonalcoholic fatty liver disease, semaglutide

## Abstract

Non-alcoholic fatty liver disease stands as the predominant cause of chronic liver disease, with its prevalence and morbidity expected to escalate significantly, leading to substantial healthcare costs and diminished health-related quality of life. It comprises a range of disease manifestations that commence with basic steatosis, involving the accumulation of lipids in hepatocytes, a distinctive histological feature. If left untreated, it often advances to non-alcoholic steatohepatitis, marked by inflammatory and/or fibrotic hepatic changes, leading to the eventual development of non-alcoholic fatty liver disease-related cirrhosis and hepatocellular carcinoma. Because of the liver's vital role in body metabolism, non-alcoholic fatty liver disease is considered both a consequence and a contributor to the metabolic abnormalities observed in the metabolic syndrome. As of date, there are no authorized pharmacological agents for non-alcoholic fatty liver disease or non-alcoholic steatohepatitis. Semaglutide, with its glycemic and weight loss advantages, could potentially offer benefits for individuals with non-alcoholic fatty liver disease. This review aims to investigate the impact of semaglutide on non-alcoholic fatty liver disease.

## Introduction and background

Diabetes mellitus (DM) is an endocrine disease that has been established to be attributed to several factors of genetic and environmental nature, the most prominent of which being an unregulated diet, physical inactivity, and tobacco smoking [1]. Global estimates of diabetic patients currently stand at around 537 million, with projections indicating a potential increase to a staggering 642 million by 2040 [2]. Furthermore, from the beginning of the millennium until 2019, type 2 diabetes mellitus (T2DM) had seen an increase in its prevalence by a factor of three, increasing from 151 million cases to 463 million globally [1].

Obesity is directly correlated with T2DM, the hallmark of which is insulin resistance (IR) [3]. In addition, obesity is a problem for the majority of those with T2DM, as it affects approximately 80% of those patients [4]. The proportion of overweight and obese people in the world has been rising for the past few decades. An estimation of as high as 30% of Americans are considered obese [4]. In terms of obesity, the adipose tissues of obese people go through a process known as hypertrophy and hyperplasia, which leads to an increase in the secretion of lipotoxic substances such as leptin, glycerol, adiponectin, and non-esterified fatty acids, along with various cytokines and chemokines that promote inflammation and cause lipotoxicity [5]. Furthermore, diabetes, cardiovascular disease, and non-alcoholic fatty liver disease (NAFLD) are all linked to obesity, which can cause IR, hypertension, and dyslipidemia [6]. It is a metabolic liver disease under which falls a wide range of progressive pathological states, beginning with mild steatosis to steatohepatitis and eventually ending in a cirrhotic liver and possibly hepatocellular carcinoma (HCC) [7]. There is a rapid rise in the prevalence of NAFLD around the world, coinciding with the epidemics of obesity and T2DM. Recent studies have revealed that a staggering 30% of the general population has evidence of NAFLD and that it affects 70-80% of obese or diabetic patients [8,9]. T2DM appears to contribute significantly to the deterioration from NAFLD to non-alcoholic steatohepatitis (NASH), cirrhosis, and possibly HCC [10]. It has become evident that NAFLD, and particularly NASH that has progressed to more advanced stages of fibrosis, also contributes to the development of the wide range of T2DM’s micro- and macrovascular complications [11]. Consequently, patients with T2DM who have NAFLD and advanced fibrosis should be monitored for signs of the disease early on [12].

Semaglutide, which functions as a glucagon-like peptide type 1 (GLP-1) agonist, is a recently available, once-weekly drug. It has recently been found to have a favorable outcome in the management of the hepatic metabolic complications that are caused by obesity, namely NAFLD and steatohepatitis, along with its use in the treatment of obesity itself [13,14]. Furthermore, this positive hepatic metabolic effect might be due to enhanced lipid degradation via the enhancement of the protein kinase AMPK (AMP-activated protein kinase) [15]. It is worthy of mention that semaglutide, as a GLP-1 receptor agonist, may influence free fatty acids by improving insulin sensitivity, inhibiting lipolysis, and affecting gastrointestinal motility. The modulation of oxidative stress (OS) may be a product of the broader metabolic improvements associated with GLP-1 receptor agonists [16]. The specific interactions and molecular pathways involved are areas of ongoing research.

In addition, the reduction of the mechanistic target of rapamycin complex 1 (mTOR) and consequently reducing the hepatic injury caused by NAFLD justify the prophylactic use of semaglutide against the disease [17]. Furthermore, the integral membrane protein α/β hydrolase domain-6 (ABHD6) has been proposed to play a role in the pathophysiology of the metabolic syndrome (MS) [18].

In fact, semaglutide was experimentally found to decrease the expression of ABHD6, which functions by hydrolysis of monoacylglycerol, and hence alleviates the degree of NAFLD.

## Review

Type 2 diabetes mellitus

Type 2 diabetes mellitus stands as the prevalent metabolic disorder, primarily distinguished by metabolic disorders, including hyperglycemia, hyperlipidemia, and IR [19]. It is linked to severe complications and concurrent health conditions, which encompass cardiovascular disease, MS, NAFLD, end-stage kidney disease, and retinopathy [20]. As of 2021, the IDF Diabetes Atlas (10th edition) has estimated that up to 537 million adults globally are diagnosed as diabetic, while 6.7 million deaths during that year have been attributed to DM. It is predicted that the number of adult patients diagnosed with diabetes will increase to 693 million by the year 2030 [21]. The main underlying factors in the development of diabetes are cellular injuries and energy metabolism dysfunction, which also play a significant role in the emergence of complications. Diagnosis is typically based on certain cutoffs of blood glucose levels. These processes impact the diabetes phenotype and subsequently contribute to the progression of both microvascular and macrovascular complications [22]. T2DM represents 90% of all documented diabetes cases and bears the primary cause for the majority of the complications associated with diabetes [23].

The pathophysiology of T2DM is significantly influenced by IR and disruptions in insulin secretion. The risk factors associated with T2DM and IR comprise obesity, higher calorie consumption, advancing age, lack of physical activity, and genetic susceptibility [24]. Moreover, a chronic state of elevated blood sugar levels leads to an intracellular rise in reactive oxygen species (ROS), potentially resulting in persistent multiorgan damage, including the liver, kidney, and brain [25]. The liver has a crucial function in the body's overall response to insulin [26]. The occurrence of NAFLD rises in individuals with diabetes [27]. Similarly, pre-existing diabetes stands as an autonomous risk factor for the advancement of NAFLD, heightened liver-related mortality, and the occurrence of HCC, as demonstrated in a prospective study [28].

Non-alcoholic fatty liver disease

**Figure 1:**
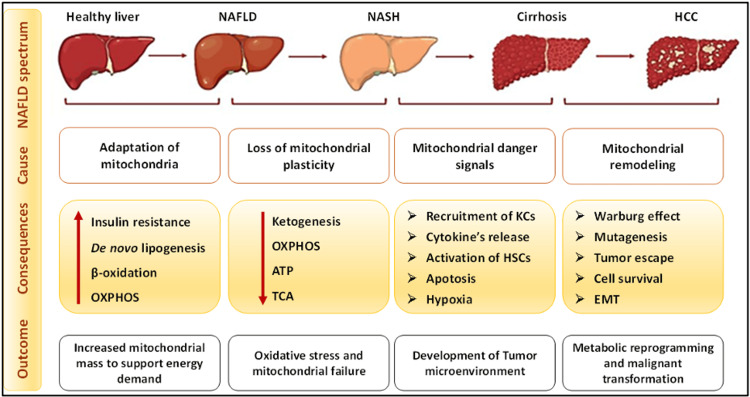
Stages and progression of liver diseases Adapted from [33]. NAFLD: non-alcoholic fatty liver disease; NASH: non-alcoholic steatohepatitis; HCC: hepatocellular carcinoma; OXPHOS: oxidative phosphorylation; ATP: adenosine triphosphate; TCA: tricarboxylic acid cycle; KCs: Kupffer cells; HSCs: hepatic stellate cells; EMT: epithelial–mesenchymal transition

Non-alcoholic fatty liver disease ranks as the most prevalent chronic hepatic condition, representing roughly 25% of all cases of chronic liver disease globally [29]. In developed countries, NAFLD stands as the leading contributor to liver disorders and abnormal liver enzymes in both Eastern and Western regions, resulting in a substantial strain on healthcare systems [30]. This disease refers to a spectrum of hepatic disorders in which deposition of lipid droplets in hepatocytes occurs due to underlying causes other than alcohol [31]. It is liable to progress to be superimposed by inflammation, which is then termed NASH. NASH can be diagnosed histopathologically when ballooning of hepatocytes and lobular inflammation is demonstrated on top of the previously established lipid accumulation. Thereafter, the pathological progression of NASH eventually leads to hepatic fibrosis, which predisposes to cirrhosis and possibly HCC (Figure 1) [32].

The pathogenesis behind NAFLD is linked to the "two-hit" theory. The initial hit is triggered by IR, stemming from an excessive influx of fatty acids into hepatocytes. The second hit involves inflammation linked to elevated insulin levels, hepatotoxicity resulting from free fatty acids, high levels of leptin, OS, and dysfunction in mitochondria [34]. The occurrence of NAFLD in individuals with T2DM was approximated to be 69% when identified through ultrasonography, compared to 87% when discovered through histopathology or MRI [35]. These assessments are anticipated due to the usual characteristics of T2DM, which involve IR, along with elevated levels of liver enzymes, triglycerides (TG), and lipids in the bloodstream [36]. Hence, NAFLD is frequently seen as an indication of MS in the liver and is commonly found as a comorbidity in individuals with T2DM [37]. Individuals with diabetes are at a significantly greater risk of developing hepatic steatosis compared to those without diabetes, with a likelihood ranging from 35% to 80% [38]. This epidemiological link indicates that NAFLD and T2DM have common causal factors, pathophysiological mechanisms, and potential treatment approaches.

Pathophysiological mechanisms shared between non-alcoholic liver disease and type 2 diabetes mellitus

**Figure 2:**
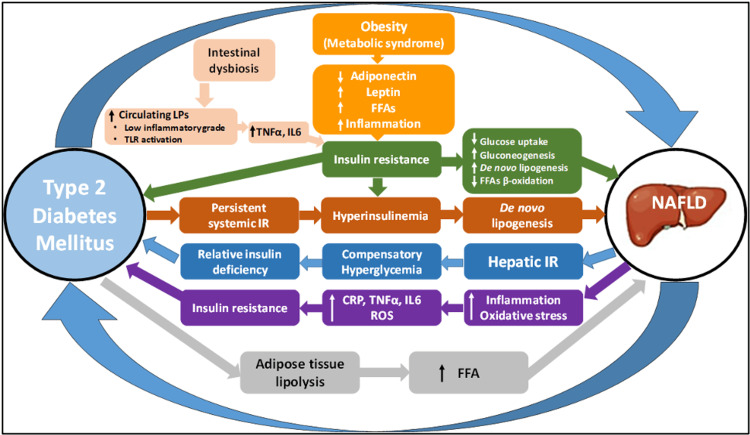
Pathophysiological mechanisms shared between NAFLD and T2DM Adapted from [39]. LPS: lipopolysaccharides; CRP: C-reactive protein; TNF-⍺: tumor necrosis factor; IL-6: interleukin-6; ROS: reactive oxygen species; NAFLD: non-alcoholic fatty liver disease; FFA: free fatty acids; TLR: toll like receptor; T2DM: type 2 diabetes mellitus

The intricate interconnection of pathophysiological processes between T2DM and NAFLD is multifaceted, encompassing various biochemical pathways and feedback mechanisms (Figure 2) [35].

Impaired glucose metabolism

Derangement in the metabolism of glucose and subsequent hyperglycemia are distinctive characteristics of T2DM. Despite elevated insulin levels, an increase in gluconeogenesis leads to both fasting and post-meal hyperglycemia [40]. Individuals with T2DM and NAFLD often exhibit liver-specific IR, where insulin is unable to effectively control the release of glucose [41]. The prolonged elevation of blood glucose levels stimulates de novo lipogenesis (DNL) through two distinct mechanisms. Firstly, it directly boosts the activity of the tricarboxylic acid cycle and the synthesis of acyl CoA, which serves as a precursor for both DNL and glucose production. Secondly, it indirectly achieves this by triggering the expression of carbohydrate-responsive element-binding protein (ChREBP) and liver X receptor-α (LXRα), which function in promoting the transcription of genes like FAS cell surface death receptor (FAS) and stearoyl-CoA desaturase 1 (SCD-1) [42]. These metabolic shifts lead to increased levels of plasma FFA and TG, along with the accumulation of ectopic fat. This, in turn, results in changes in insulin function, reduced ATP synthesis in muscles, diminished production of nitric oxide (NO), and reduced insulin-stimulated activation of phosphoinositol-3 kinase (PI3K), pyruvate dehydrogenase kinase isozyme 1, and endothelial nitric oxide synthase (eNOS). Furthermore, the persistent increase in blood glucose levels leads to progressive damage to the beta cells, an increase in the release of OS and ROS, cytoplasmic DNA fragmentation, alterations in mitochondrial structure, and the triggering of proapoptotic pathways [42].

Insulin resistance

Individuals with IR who have NAFLD exhibit diminished insulin sensitivity not just in muscle but also in the adipose tissue and liver [43]. In scenarios of IR, adipose tissue progressively becomes resistant to the insulin's ability to inhibit fat release, leading to an elevated release of fatty acids [44]. IR is characterized by higher insulin levels, which, when combined with elevated lipolysis and/or increased dietary fat intake, encourage the synthesis of TG in the liver [45]. Hepatic IR, induced by the activation of protein kinase C epsilon (PKC𝜀) through diacylglycerol, could be the pivotal pathological connection between NAFLD and T2DM [46].

Oxidative stress

In the liver, diabetes is regarded as the primary factor responsible for triggering OS, which elevates the concentration of ROS within the tissue. This, in turn, significantly reduces the levels of antioxidants, leading to elevations in lipid peroxidation and protein oxidation [47]. The body consistently generates free radicals through routine metabolic processes and interactions with environmental factors. In normal circumstances, antioxidants shield the body from the harmful effects of free radical production in vivo. OS arises from a disproportion between systems that generate radicals and those that effectively neutralize them, resulting in either an escalation of free radical production, a reduction in antioxidant activity, or both. In diabetes, protein glycation and glucose autoxidation have the potential to induce the generation of free radicals and accelerate the process of lipid peroxidation. Furthermore, the deficiencies in the body's antioxidant defenses become evident in diabetes. This includes alterations in antioxidant enzyme function, disruptions in glutathione metabolism, and diminished levels of ascorbic acid [48].

The extent of the progression from NAFLD to steatohepatitis depends on the extent of oxidative damage occurring in the liver [47]. In investigating the connection between insulin and leptin resistance in hepatic steatosis, Zhang and co-workers found that IR occurs initially, followed by the development of leptin resistance, and, subsequently, steatosis approximately two to three weeks later [49]. However, the link between leptin and hepatic steatosis has generated differing conclusions, despite the frequent occurrence of leptin resistance in obese individuals. This resistance is a significant contributor to the accumulation of hepatic fats and, consequently, the development of steatosis [50].

Inflammation

Inflammatory pathways are integral to the development of IR. Notably, chronic inflammation and a variety of mediators are released by various immune cells and adipocytes, most notably IL-6, TNF- α, and IL-1 [51]. Inflammation is linked with heightened OS and mitochondrial dysfunction. Hepatocellular inflammation involves various inflammatory cells; Kupffer cells, specifically, are integral to the development and advancement of NAFLD. They regulate the storage of TG in the liver, facilitate the inflammatory process, contribute to the process of lipid peroxidation, generate ROS, and activate nuclear transcription factors like NF-κB. These factors control the release of pro-inflammatory cytokines, namely TNF-α and TGF-β, ultimately leading to injury in liver tissue [35]. Moreover, FFAs act as the main instigators of inflammation by promoting mitochondrial lipid oxidation, elevating ROS, and augmenting microsomal cytochromes like CYP4A1 and CYP2E1 [52]. Lipid peroxidation is a disruptive process that targets the polyunsaturated fatty acids found in cell membranes. It generates compounds like malondialdehyde and 4-hydroxy-2-nonenal, which can diffuse from the cell and impact areas distant from the initial site of oxidation, making them crucial cytotoxic mediators. In addition, these compounds aid in the chemotaxis of neutrophils and have the potential to activate hepatic stellate cells, leading to increased collagen expression and the accumulation of fibrous tissue [51]. Additionally, individuals diagnosed with T2DM experience heightened systemic inflammation marked by increased levels of C-reactive protein, IL-1β, and IL-6 [53]. The excess accumulation of body fat frequently observed in individuals with T2DM is associated with the heightened production of proinflammatory cytokines by adipose tissue [54]. It was found that the mix of cytokines in the liver significantly contributes to the emergence of various aspects of human NAFLD, such as inflammation, fibrosis, and the formation of tumors [55].

AMP-activated protein kinase

AMP-activated protein kinase (AMPK) consists of a trimeric assembly that includes three subunits: an α subunit weighing 63 kDa, a β subunit weighing 38 kDa, and a γ subunit also weighing 38 kDa, wherein the α subunit is the catalytic portion, whereas the β and γ subunits possess regulatory functions [56]. The α subunit consists of three domains spanning from the N-terminal to the C-terminal: a kinase domain, a self-inhibitory domain that reduces the activity of AMPK at low levels of AMP, and a carboxy-terminal domain [57]. Furthermore, alterations in the intracellular ratio of AMP to ATP significantly influenced AMPK activity. When the levels of AMP/ATP increased, it led to the activation of AMPK, which, in turn, inhibited lipogenesis and enhanced the oxidation of fatty acids [58]. In mammals, these three subunits are present in seven different forms, specifically α1 and α2, β1 and β2, and γ1, γ2, and γ3 [59]. The predominant expression of subunits α1, α2, γ1, and γ2 occurs in the liver [57]. AMPK, a critical component in metabolic regulation, acts as an indicator of OS to the cells in response to OS and energy deficits within the body. It then governs target proteins through phosphorylation, impacting lipid metabolism [60]. Prior research has established that upon activation, AMPK has the ability to control lipid metabolism at the cellular level via the phosphorylation of several proteins that impact the synthesis of fatty acids, cholesterol, and the oxidation of fatty acids. As an example, AMPK, through phosphorylation, suppressed the function of 3-hydroxy-3-methylglutaryl-CoA reductase (HMGR), a pivotal player in the regulation of cholesterol synthesis [61]. Simultaneously, AMPK has the capacity to phosphorylate and activate hormone-sensitive lipase (HSL), subsequently enhancing the breakdown of fatty acylglycerol and cholesterol lipids [62]. Furthermore, certain researchers identified possible mechanisms by which AMPK diminishes the buildup of fat in the liver [63,64]. AMPK in its active state is capable of phosphorylating and subsequently deactivating acetyl-CoA-carboxylase (ACC), thereby preventing its dimerization and consequently reducing the synthesis of fatty acids. Malonyl-CoA serves as a synthetic precursor for fatty acids and simultaneously inhibits the activity of carnitine acyltransferase 1 (CPT1). AMPK aids in the phosphorylation of ACC, rendering it inactive, which in turn diminishes the production of malonyl-CoA. This reduction enhances the expression of CPT1, thereby boosting the oxidation of fatty acids. To summarize, AMPK has the capability to phosphorylate essential downstream proteins, decreasing the buildup of lipids, enhancing the oxidation of fatty acids, and impeding the production of cholesterol and fatty acids. Consequently, the AMPK pathway holds potential as a potential therapeutic approach in the treatment of NAFLD.

Mechanistic target of rapamycin

The mechanistic target of rapamycin is a serine/threonine protein kinase possessing a molecular weight of 289 kDa, belonging to the phosphatidylinositol 3-kinase-related kinase (PIKK) family. It operates within two distinct complexes: mTORC1 and mTORC2 [65]. The mTORC1 pathway contributes to both lipogenesis and easing NAFLD by regulating the autophagy process. The activity of the mTOR pathway is affected by a variety of triggers, including a high-fat diet, excessive intake of processed carbohydrates, and elevated insulin levels. These factors regulate the activation of mTOR, often via the stimulation of the PI3K/AKT axis [66]. The mTOR pathway is sensitive to different sources of energy, including carbohydrates, lipids, proteins, and nucleic acids. It achieves this via the activation of mTOR complexes (mTORC1 and mTORC2). Consequently, mTOR is vital in controlling many metabolic processes, in addition to cellular proliferation and differentiation [67]. Thus, the mTOR complex is crucial in the control of nutrient availability and growth factors influencing various mechanisms and genes [68]. The synthesis of fatty acids and triacylglycerol in the liver is significantly regulated by DNL-induced Sterol Regulatory Element-Binding Protein 1 (SREBP1), a crucial factor in nutritional control. The mTORC1 pathway regulates anabolism and catabolism in accordance with fluctuating physiological nutrient levels [68]. Additionally, mTORC1 is involved in regulating DNL by boosting the transcription of SREBP1, which contributes to the development of NAFLD [69]. In situations of excessive nutrient intake when the liver is unable to completely metabolize the surplus dietary nutrients, the transformation of DNL into triacylglycerol becomes crucial. Subsequently, mTORC2 oversees the regulation of ChREBP1, leading to hepatic insulin resistance and DNL, while also promoting the accumulation of fat in the liver, contributing to NAFLD. Reducing ChREBP1 expression is necessary to eliminate the RICTOR subunit (the RPTOR independent companion of mTORC2) from mTORC2, which typically occurs during fasting [70-72].

α/β hydrolase domain-6

The α/β hydrolase domain-6 is a newly identified integral membrane protein that can break down monoacylglycerol [73].

Earlier research has indicated that ABHD6 is mainly responsible for controlling endocannabinoid signaling within the brain [74]. Nevertheless, the latest findings implicate ABHD6 in the development of different metabolic conditions, including MS, disorders in insulin secretion, obesity, and T2DM [75-77]. Notably, ABHD6 is widely expressed, with its presence detected in various tissues, including the liver, kidney, small intestine, and white adipose tissue [78]. When antisense oligonucleotide (ASO) was used to suppress the expression of ABHD6 in peripheral tissues, it resulted in weight loss, decreased hepatic steatosis, and improvement in various metabolic derangements in mice on a high-fat diet [75]. Moreover, the increased browning of adipose tissue and improved function of brown adipose tissue in ABHD6-knockout mice are, to some extent, a result of the buildup of 1-monoacylglycerol, which activates both PPARα and peroxisome proliferator-activated receptor gamma (PPARγ) [77]. In summary, suppressing ABHD6 activity may hold promise for potential therapeutic applications in managing MS, obesity, and NAFLD.

Management of non-alcoholic fatty liver disease

Lifestyle alterations continue to be the fundamental approach in managing NAFLD and are recommended for all individuals affected by the condition [79]. The evidence indicates that a Mediterranean diet is among the recommended dietary patterns, coupled with a focus on reducing calories to manage a 7-10% reduction in baseline body weight [80]. According to the current guidelines provided by the American College of Sports Medicine (ACSM) and the World Health Organization (WHO), it's recommended to incorporate exercise along with dieting, considering individual cardiovascular risk factors and any associated health conditions [81]. The complexity of managing NAFLD is due to the lack of well-established, effective disease-modifying treatment options [82]. The management strategies have an indirect impact by enhancing IR and glycemic control, which consequently aids in the treatment of T2DM. Therefore, pharmaceutical treatment should be limited to individuals with the most substantial risk of disease advancement in NAFLD. Consequently, the focus is on managing associated or coexisting conditions (such as diabetes, obesity, and NAFLD) to regulate the patient’s glycemic levels, hepatic function, and lipid profile [83].

Pharmacological treatment is advised for patients who fail to reach their targeted body weight parameters and for patients whose disease has progressed to NASH and a stage of fibrosis of 2 or higher (F2) as confirmed by a biopsy [84]. The Greek Atorvastatin and Coronary Heart Disease Evaluation (GREACE) trial examined the safety of statins in NAFLD. The author's findings indicated that statin treatment is well-tolerated and can enhance liver function tests while decreasing the risk of cardiovascular complications in individuals with mildly to moderately abnormal liver tests that may be related to NAFLD [85]. While hyperinsulinemia linked to IR can harm the liver, the administration of exogenous insulin in patients with T2DM can be beneficial. Research demonstrated that T2DM patients, who had inadequate control using oral antidiabetic medications, experienced a reduction in hepatic fat by 12.6% to 9.9% on proton magnetic resonance spectroscopy (MRS) after a 12-week treatment with insulin glargine. Simultaneously, there was an enhancement in glycated hemoglobin (HbA1c) from 7.9% to 7.2% [86].

Metformin remains a first-line agent in the treatment of T2DM [82]. It reduces body fat, enhances hepatic insulin sensitivity, promotes fatty acid oxidation, and lowers DNL [87,88]. It has not been approved for the treatment of NAFLD in the absence of diabetes [82]. In individuals diagnosed with NAFLD, metformin led to reduced levels of serum aminotransferases and enhanced IR. Nevertheless, it did not induce any changes in liver histology [89]. Furthermore, a recent meta-analysis suggested that metformin could potentially serve as a viable medication for managing NAFLD [90]. Moreover, the use of sulfonylureas for NAFLD induced by T2DM has not yet been supported by prospective studies [91]. Typically, both conditions present OS, which may be alleviated by using a dosage of 800 IU/day of vitamin E for a duration of 96 weeks. This treatment regimen can enhance liver function, steatosis, inflammation, and ballooning (with the exception of fibrosis) and result in NASH resolution in 43% of patients [92]. Pentoxifylline, a non-specific phosphodiesterase inhibitor, plays a role in reducing inflammatory mediators like TNF-α [93]. There have been conflicting findings regarding changes in plasma aminotransferases and imaging for hepatic steatosis, as some studies have demonstrated improvements while others have shown no significant changes [94]. Thiazolidinediones enhance insulin sensitivity in adipose tissue by activating PPARγ, which results in increased fatty acid uptake and storage [95]. An elevation in adiponectin levels accompanies the improvement of proinflammatory adipokines, leading to a reduction in gluconeogenesis and enhancement in the influx of fatty acids and insulin sensitivity [96]. Additionally, they facilitate the restoration of typical adipose tissue functions and contribute to the reduction of fat accumulation in the liver [97]. Presently, there is inadequate research evidence available to distinguish the use of various dipeptidyl peptidase IV (DDP-IV) inhibitors in individuals with both diabetes and NAFLD. Furthermore, they are relatively contraindicated to be used in patients with severe liver derangement [98]. Moreover, sodium glucose cotransporter 2 inhibitors (SGLT2) are often useful in ameliorating steatosis and inflammation. Glycosuria, through its induction of negative energy balance and the shift toward utilizing lipids as an energy source, can lead to halting the progression of steatosis and fibrosis [82]. More research studies are needed before these agents can be implemented clinically. In March 2024, resmetirom (a thyroid hormone receptor-β agonist) received accelerated approval in the USA for use with diet and exercise to treat adults with noncirrhotic NASH with moderate to advanced liver fibrosis. The medication is also under regulatory review in the European Union for the treatment of NASH [99]. Interestingly, animal studies have indicated that analogs of glucagon-like peptide-1 (GLP-1) help alleviate hepatic steatosis and steatohepatitis [98,100,101].

Glucagon-like peptide-1 receptor agonists

Glucagon-like peptide-1 (GLP-1) receptor agonists could present favorable therapeutic possibilities in managing NAFLD due to their advantageous impacts on blood sugar levels and weight reduction [102,103]. Human hepatocytes have been observed to contain GLP-1 receptors, and there is a hypothesis suggesting that activating these receptors with GLP-1 receptor agonists may yield beneficial outcomes on hepatic steatosis, fatty acid oxidation, lipotoxicity, and the cytokines linked to liver inflammation and fibrosis [104]. Additionally, GLP-1 receptor agonists could potentially offer indirect beneficial effects on the liver by enhancing insulin release in accordance with elevated blood sugar levels, reducing the release of glucagon, prolonging gastric emptying, and leading to substantial weight loss [14].

Out of the GLP-1 receptor agonists, semaglutide has displayed the most significant advantages with regard to optimizing glycemic control and weight loss [105]. In a recently conducted phase three clinical trial involving overweight and obese individuals, semaglutide exhibited a substantial reduction in body weight, amounting to 14.9%, in contrast to the 2.4% decrease observed in the placebo group [106]. Furthermore, semaglutide has demonstrated decreased occurrences of cardiovascular and renal complications in T2DM patients [107-110]. It has subsequently gained approval for the management of T2DM and long-term weight control.

Semaglutide

Semaglutide (Ozempic®; Novo Nordisk A/S, Denmark) is an analog of GLP-1 approved for once-weekly subcutaneous treatment of T2DM in many countries [111]. The extensive SUSTAIN program revealed that once-weekly subcutaneous semaglutide displayed effective glycemic control compared to various comparator treatments. It also offered supplementary clinical advantages, including weight reduction, decreased cardiovascular risk, and lowered systolic blood pressure, all with minimal incidence of hypoglycemia [112].In addition, the Semaglutide Treatment Effect in People with Obesity (STEP) trials demonstrated that a high-dose, once-weekly 2.4 mg semaglutide effectively reduces body weight in individuals with obesity. Although semaglutide was associated with a higher incidence of gastrointestinal side effects, it was generally safe and well tolerated [113]. In January 2020, Ozempic received FDA approval for its use in adults diagnosed with type 2 diabetes [111]. Recent studies indicate that treatment with semaglutide has shown notable enhancements in liver enzymes, decreased liver stiffness, and improved metabolic parameters among individuals with NAFLD/NASH (Table 1). 

**Table 1:** Studies on weekly subcutaneous semaglutide for NAFLD/NASH NAFLD: non-alcoholic fatty liver disease; NASH: non-alcoholic steatohepatitis

Reference	Type of the study	Medication	Number of patients and duration of the treatment	Effects
Davies et al. (2021) [114]	Double-blind, double-dummy phase 3 superiority study	Once-weekly subcutaneous administration of semaglutide 2.4 mg versus semaglutide 1.0 mg versus placebo.	1164 for 68 weeks	Semaglutide notably lowered ALT and C-reactive protein levels involving individuals with type 2 diabetes and/or obesity.
Okamoto et al. (2021) [115]	Retrospective, single-center, cohort study	Semaglutide 0.25 mg was administered once weekly, and the dosage was increased to 0.5 mg once weekly after four weeks, or to 1.0 mg once weekly if the 0.5 mg dose for ≥4 weeks proved insufficient in terms of efficacy.	50 for six months	Semaglutide seems to be more effective in treating type 2 diabetes compared to other GLP-1 RAs. Liver-related parameters showed significant improvement after six months of treatment.
Volpe et al. (2022) [116]	Prospective, single-arm, real-life study	Semaglutide administered at doses ranging from 0.25 mg to 1 mg weekly.	48 for 52 weeks	Semaglutide improved glucose control and body composition, as well as enhanced the clinical presentation and reduced the severity of NAFLD in patients with type 2 diabetes.
Schattenberg et al. (2023) [117]	Randomized, placebo-controlled trials	Once-weekly subcutaneous semaglutide (2.4 mg in STEP 1 and 1.0 mg or 2.4 mg in STEP 2)	STEP 1:1307 STEP 2: 643 for 68 weeks	Semaglutide showed a favorable impact on NASH components in overweight/obese patients with or without type 2 diabetes.

Glucagon-like peptide type 1 receptor agonists could potentially exert beneficial effects on oxidative stress through direct inhibition of the generation of OS through the cAMP-PKA pathway activated by the GLP-1 receptor, which in turn hinders oxidative stress production derived from NADPH oxidase [118]. Moreover, in experimental animal models, atherosclerotic lesion progression showed more favorable outcomes with increased circulating concentrations of GLP-1, attributed to the suppression of oxidative stress [16]. Furthermore, semaglutide suppressed the increased expression of pro-inflammatory factors, namely TNF-α and IL-6, and reduced overall fat accumulation in the liver [119].

Semaglutide (C187H291N45O59), which has a mass of 4113.58 g/mol, possesses a peptide structure composed of 31 amino acids. This structure is 94% similar to the natural GLP-1, which aims to reduce potential immunogenic responses [120]. The eighth position's alanine residue is replaced by Aib (2 aminoisobutyric acid), preventing the DDP-4 enzymes from degrading semaglutide. Similar to liraglutide, semaglutide also features a substitution of the lysine residue at the 34th position found in native GLP-1 with arginine. The replacement of arginine at the 34th position was crucial in the creation of the GLP-1 analog using the asemi-recombinant process [121]. The lysine residue at the 26th position was modified by acylation to facilitate its attachment to a C18 fatty diacid using a hydrophilic linker called "γGlu-2xOEG". This modification extended the half-life of the compound in the body by increasing its binding to albumin and reducing renal clearance [122].

Semaglutide enhances the effectiveness of incretin function through the activation of GLP-1 receptors. It acts through various mechanisms, including increased insulin secretion (dependent on glucose levels), suppression of glucagon secretion, and inhibition of gluconeogenesis in the liver, resulting in lower fasting and postprandial blood glucose levels [122]. Semaglutide has exhibited a promising proinsulin to insulin ratio, indicating enhanced efficiency in beta-cell function and increased insulin production [123,124]. It also showed enhanced insulin sensitivity, potentially attributed to an overall decrease in body weight [125]. Additionally, semaglutide results in weight reduction, a product of decreased caloric consumption and delayed gastric motility [126]. Semaglutide has an extended half-life of approximately one week, which makes it optimal for once-weekly injections [127,128]. In disease-free Caucasian and Japanese individuals, the once-weekly administration of semaglutide leads to dose-dependent increases in exposure for both 0.5 mg and 1 mg doses [129]. Clinical studies in pharmacology and population pharmacokinetics analyses from phase 3a trials have indicated that no dose adjustment is necessary based on renal or hepatic impairment or ethnicity [130,131]. Additionally, the population pharmacokinetic analysis revealed that the most significant indicator of semaglutide exposure is body weight, as overweight and obese individuals tend to exhibit lower semaglutide exposure [130,132]. It was found that drug interactions have indicated that there is no need for dosage adjustments with commonly prescribed oral medications when taken alongside once-weekly subcutaneous semaglutide [133].

While generally well tolerated, semaglutide exhibits gastrointestinal upset of varying severity, such as nausea, vomiting, altered bowel movements, and indigestion. These symptoms are typically dose-dependent and tend to diminish within two weeks of starting treatment [134]. Less common adverse effects of semaglutide include nasopharyngitis, headaches, infections in the urinary and upper respiratory tracts, as well as elevated levels of pancreatic enzymes (amylase and lipase) [112,135-137]. In some instances, isolated cases of acute pancreatitis have been documented, necessitating the cessation of treatment. Episodes of hypoglycemia are rare with semaglutide. However, when used with insulin analogs or other medications that lower blood sugar, a dose reduction is typically required [121]. Besides these, extremely uncommon instances of temporary reactions at the injection site have also been documented [123]. Similar to other GLP-1 agonists, semaglutide exhibits some infrequent adverse effects, including tachycardia, in addition to cases of cholelithiasis and cholecystitis [126].

## Conclusions

Treatment with semaglutide showed notable enhancements in liver enzymes, decreased liver stiffness, and improved metabolic parameters among individuals with NAFLD/NASH. Hence, it could possess hepatoprotective effects in NAFLD patients associated with T2DM. Nevertheless, there are concerns regarding potential gastrointestinal side effects. Further experimental studies are recommended to delve into the detailed molecular mechanisms through which semaglutide affects NAFLD as well as evaluate the occurrence rates of side effects associated with semaglutide (safety of semaglutide). Moreover, clinical studies are advised to determine the clinical effectiveness of semaglutide treatment in individuals with various metabolic conditions, such as NAFLD and liver fibrosis.
